# Advances in reprogramming of energy metabolism in tumor T cells

**DOI:** 10.3389/fimmu.2024.1347181

**Published:** 2024-02-13

**Authors:** Liu Xuekai, Song Yan, Chu Jian, Song Yifei, Wu Xinyue, Zhang Wenyuan, Han Shuwen, Yang Xi

**Affiliations:** ^1^ Department of Clinical Laboratory, Aerospace Center Hospital, Beijing, China; ^2^ Department of Medical Oncology, Huzhou Central Hospital, Affiliated Central Hospital Huzhou University, Huzhou, China; ^3^ Department of Gastroenterology, Fifth School of Clinical Medicine of Zhejiang Chinese Medical University (Huzhou Central Hospital), Huzhou, China; ^4^ Department of Key Laboratory of Multiomics Research and Clinical Transformation of Digestive Cancer, Huzhou, China; ^5^ Department of Gynecology, Heyuan Hospital of Traditional Chinese Medicine, Heyuan, China

**Keywords:** metabolic reprogramming, T cells, immunotherapy, immune microenvironment, energy metabolism

## Abstract

Cancer is a leading cause of human death worldwide, and the modulation of the metabolic properties of T cells employed in cancer immunotherapy holds great promise for combating cancer. As a crucial factor, energy metabolism influences the activation, proliferation, and function of T cells, and thus metabolic reprogramming of T cells is a unique research perspective in cancer immunology. Special conditions within the tumor microenvironment and high-energy demands lead to alterations in the energy metabolism of T cells. In-depth research on the reprogramming of energy metabolism in T cells can reveal the mechanisms underlying tumor immune tolerance and provide important clues for the development of new tumor immunotherapy strategies as well. Therefore, the study of T cell energy metabolism has important clinical significance and potential applications. In the study, the current achievements in the reprogramming of T cell energy metabolism were reviewed. Then, the influencing factors associated with T cell energy metabolism were introduced. In addition, T cell energy metabolism in cancer immunotherapy was summarized, which highlighted its potential significance in enhancing T cell function and therapeutic outcomes. In summary, energy exhaustion of T cells leads to functional exhaustion, thus resulting in immune evasion by cancer cells. A better understanding of reprogramming of T cell energy metabolism may enable immunotherapy to combat cancer and holds promise for optimizing and enhancing existing therapeutic approaches.

## Introduction

Cancer is one of the leading causes of death globally and affects a large number of people. Cancer treatment has progressed from traditional approaches, such as surgical resection, radiotherapy, and chemotherapy, to immunotherapies, such as immune checkpoint inhibitors (ICIs), chimeric antigen receptor T cells (CAR-T cells), and cytokine therapies (1–3). Immunotherapy refers to the use of the body’s immune system to treat cancer. Different from traditional therapies, immunotherapy employs a variety of immune cells (such as T cells, natural killer (NK) cells, and dendritic cells), cytokines, and chemokines [such as C-X-C motif chemokine ligand 12 (CXCL12), CXCL10, and C-C motif chemokine ligand 5 (CCL5)] to remodel the tumor microenvironment (TME), which can yield potent effects and aid in preventing cancer recurrence (4). The advent of immunotherapy has sparked a paradigm shift in the standards and approaches of cancer treatment. Although immunotherapy is a promising treatment, a significant proportion of patients exhibit limited or no response to immunotherapy (5, 6). Therefore, a better understanding of the key components of TME may help refine current immunotherapeutic strategies.

TME consists of a heterogeneous milieu of tumor and immune cells, and has been shown to participate in tumor progression and affect immunotherapy response (7). As an important component of TME, T lymphocytes (T cells) are not only key cells of cellular immunity, but also an important focus in anti-tumor responses (8). T cells derived from the bone marrow lymphoid stem differentiate into mature cells in the thymus. Naïve T cells can be divided into two main subsets, namely CD4+ and CD8+ T cells, which have patrol and surveillance functions (9). Once activated by antigen-presenting cells (APCs), CD4+ T cells can rapidly differentiate into different subtypes: CD4+ effector T cell (Teff) exhibits anti-tumor activity, and regulatory T cell (Treg) has immunosuppressive and tumor-promoting effects (10). CD8+ T cells can differentiate into CD8+ Teff cells (also known as cytotoxic T cells) and memory T cells (Tmem) (11). CD8+ Teff cells directly kill tumor cells by promoting apoptosis and cytokine secretion. Tmem cells can recognize immune memory antigens and rapidly exert their tumor cell-killing function, which is crucial for long-term survival of the host. In the final stages of tumor development, CD8+ T cells are active and exert anti-tumor effects (9). In addition, T cells participate in the immune regulation of almost all cancers, including colorectal cancer (CRC), and are considered as critical determinants of clinical outcomes (12, 13). A range of T cell-based therapies have demonstrated efficacy in inducing complete and long-lasting responses in patients with various cancer types (14–16). The function of immunosuppressive checkpoints in T cells, including programmed cell death protein (PD-1) and cytotoxic T lymphocyte (CTL)-associated protein 4 (CTLA-4), provides a basis for tumor immunotherapy. These checkpoints regulate T cell activity and play a role in preventing excessive immune responses. However, some tumor cells can exploit these checkpoints to evade immune detection and attack (17–19).

Immune cells share similarities with tumor cells in terms of their involvement in metabolic regulation to maintain cell proliferation and survival (20). Following activation, T cells may undergo metabolic reprogramming characterized by a switch from oxidative metabolism to aerobic glycolysis, also known as Warburg effect (21, 22). This metabolic shift requires precise metabolic rewiring to enable T cells to fulfill the increased energy requirements for their proliferation and effector functions (21, 22). Chang et al. suggested that cell can use either mitochondrial metabolism or glycolysis for ATP generation. When glycolysis in T cells is blocked, their ability to produce iinterferon (IFN)-γ is impaired, and then anti-tumor immune response is reduced (23). Therefore, adjusting the metabolic processes and functions of T cells has potential for improving the efficacy of tumor immunotherapy (24, 25). Mitochondria are dynamic and interconnected organelles that are core regulators of metabolic reprogramming and play crucial roles in controlling the activation and function of immune cells such as T cells (26). Ron-Harel et al. also revealed that during T cell activation, mitochondrial proteome remodeling generates specialized mitochondria with enhanced one-carbon metabolism, which is critical for T cell activation and survival (27). However, T cell energy metabolism in TME and its effects on tumors have not been comprehensively reported.

In this review, the recent progress in research on T cell energy metabolic reprogramming is described, including T cell energy metabolism and its role in regulating T cell function, influencing factors, and T cell energy metabolism in cancer immunotherapy. A better understanding of T cell metabolic programming in TME holds promise for enhancing the anti-tumor immune response and improving the efficacy of tumor immunotherapy.

## Role in regulating T cell function

Metabolic reprogramming has recently become a rapidly growing field in tumor research, and it is not limited to tumor cells, but can also occur in immune cells (28). Among the various immune cells with different functions, T cells are the main cell type that exert anti-tumor effects in the adaptive immune stage (29). The proliferation and differentiation of T cells are strongly influenced by dynamic changes in their metabolism (30). T cells in the quiescent phase have low metabolic requirements, and low levels of oxidative phosphorylation (OXPHOS) can meet their growth and survival needs. When they encounter antigens, quiescent T cells are activated and require energy to undergo metabolic reprogramming to support their anti-tumor functions (31, 32). Similar to rapidly proliferating tumor cells, the metabolism of T cells shifts from low levels of OXPHOS to high levels of aerobic glycolysis and glutamine metabolism (33).

Mitochondria are the major sites for cellular energy production. Mitochondrial metabolism is the core of the cellular metabolic network, encompassing processes such as OXPHOS, the tricarboxylic acid (TCA) cycle, and amino acid metabolism, all of which occur within the mitochondria (34). Abnormal levels of reactive oxygen species (ROS) in tumor cells can damage mitochondrial DNA (mtDNA), thereby affecting the normal function of the mitochondrial membrane respiratory chain and adenosine triphosphate (ATP) production system (35). The rapid increase in ROS accelerates the destruction of mitochondrial function, thus resulting in tumor cells that obtain ATP through glycolysis and metabolic reprogramming (36). Moreover, prolonged antigen exposure, hypoxia within the TME, or inhibitory signaling can induce various mitochondrial changes in T cells, such as aberrant localization, altered morphology, disrupted structure, and/or perturbed membrane potential (ΔΨm) (37, 38). These modifications are closely linked to functional deterioration, such as suppressed mitochondrial biogenesis, diminished ATP generation, mitochondrial reactive oxygen species (mtROS) accumulation, disrupted mitophagy, and impaired OXPHOS and fatty acid oxidation (FAO) (26), thereby resulting in an inability to fulfill energy requirements and the occurrence of T cell exhaustion. Mitochondrial morphology controls T cell metabolism and can destroy tumor cells by enhancing T cell recognition. Activated Teff cells possess fragmented mitochondria, whereas Tmem cells maintain the mitochondria in a fused network. By modulating cristae morphology, fusion in Tmem cells regulates the association of the electron transport chain (ETC) complex, which promotes OXPHOS and FAO. Conversely, fission in Teff cells results in expansion of cristae, impairment of ETC efficiency, and preference of aerobic glycolysis (39). Furthermore, mitochondria contain large amounts of Ca2+ and control the absorption and uptake of calcium ions within the cell. Taken together, these results indicate that mitochondria play a key role in controlling T cell activation and function.

## Various pathways associated with T cell energy metabolism

### Glycolytic pathway

Mitochondria are hubs of cellular metabolism, including glucose, amino acid, and fatty acid metabolism (40). As the main pathway of energy metabolism in tumor cells, glycolysis is crucial for the proliferation and functional activities of immune cells (41). Upon T cell activation, there is a shift in their metabolism from a quiescent state, primarily relying on OXPHOS, to an activated state characterized by increased glycolysis, which allows for the swift production of ATP and metabolic intermediates necessary for cell proliferation and effector molecule synthesis (21, 22). It has been reported that the naïve T cells rely on OXPHOS to maintain energy requirements, while activated T cells consume a large amount of glucose through aerobic glycolysis to exert anti-tumor effects (42, 43). HIF-1α is required for effector states in CD8+T cells, and the loss of HIF-1α in CD8+T cells reduces tumor invasion and tumor cell killing and alters tumor vascularization (44). Mitochondrial dysfunction causes redox stress, which inhibits proteasomal degradation of Hypoxia-inducible factor-1α (HIF-1α) and promotes transcription and metabolic reprogramming of Tpex cells to become exhausted T cells (45). In addition, MYC plays a key role in regulating the response of HIF-1α to hypoxia, and the MYC signaling pathway is uncoupled from the increased transcription of HIF-dependent glycolytic genes in glycolytic flux during hypoxia (46). In CD4+T cells, the loss of HIF-1α or HIF-1α and HIF-2α also impairs essential functions during antibody response. Glycolysis increases the expression of T helper cytokines, and HIF promotes glycolysis in T helper cells through TCR or cytokine stimulation (47). Solute carrier (SLC) plays an important physiological function in cells. Increased glucose uptake mediated by GLUT1 and GLUT3 transporters maintains T cell activation and promotes differentiation (48). After entering the site of inflammation, CD4+ and CD8+ T cell subsets internalize high levels of lactic acid via SLC15A2 and MCT1 (SLC16A1), respectively (49, 50). Macintyre AN et al. ‘s findings suggest that Glut1 deficiency leads to impaired glucose uptake and glycolysis within CD4 T cells, thereby reducing the survival and differentiation ability of T effector cells, as well as the ability to induce inflammatory diseases *in vivo*. However, the function of Tregs does not appear to be affected by the Glut1 defect (48). In addition, metabolites produced during glycolysis also participate in the activation and effector function of T cells through other ways. Teff cells up-regulate specific glycolytic pathways to achieve rapid proliferation and play anti-tumor effect, including aerobic glycolysis, pentose phosphate pathway (PPP), hexosamine biosynthetic pathway (HBP), and TCA cycle support (51). CD8 T cells are an important part of human adaptive immune response, and changes in glycolysis have important effects on their activation and function. Activation of CD8 T cells by TCR leads to the transfer of cellular metabolic level to glycolysis, while the synergistic stimulation of CD28 further increases the glycolysis level of CD8 T cells to support subsequent proliferation and differentiation (52). The induction of high glycolytic activity is conducive to the differentiation of CD8 T cells into effector cells, but it can seriously impair the survival ability of Tmem cells (53, 54).

### Amino acid metabolic pathway

In addition to glycolysis, the massive proliferation of activated T cells relies primarily on amino acid metabolism to support protein and nucleotide synthesis (55). Glutamine metabolism supplies carbon and nitrogen necessary for the synthesis of amino acids, nucleic acids, and lipids. The lack of glutamine inhibits T cell proliferation and cytokine production, which implies that the restoring high levels of glutamine to the TME can enhance T cell killing of tumor cells (56).

Besides, arginine is a precursor in the biosynthesis of many macromolecules, including proteins, creatine, and polyamines (57–59). As arginine deprivation can lead to cell cycle arrest, arginine is important for T cell proliferation (60). Elevated L-arginine levels induced a shift from glycolysis to oxidative phosphorylation in activated T cells and promoted the production of central memory-like cells with higher viability. Intracellular L-arginine concentration can directly affect the metabolic adaptability and viability of T cells, which also reveals the importance of its anti-tumor response (61).

The presence of extracellular methionine influences the epigenetic programming responsible for shaping the destiny of CD8+ T cells and synergizes with serine (which can be synthesized internally from glucose or obtained externally) to stimulate one-carbon metabolism (62–64). Lysine catabolism can affect CD8+ T cells to reprogram tumor immunity via histone crotonylation (65). Amino acids, particularly arginine and leucine, signal to allow TCR-induced mTORC1 activation and subsequent changes in mitochondrial metabolism in Treg cells via RagA/B and Rheb1/2 (66). SLC1A5 or SLC38A1 cotransports glutamine and leucine exchanged via the SLC7A5-SLC3A2 complex can promote mTORC1 activation through direct and indirect mechanisms, thereby regulating T cell metabolism (67). Tryptophan is an essential amino acid and factor in determining the strength and effectiveness of T cell responses in TME. Tryptophan can directly activate cytoplasmic transcription factor-aryl hydrocarbon receptor (AhR) by producing kynurenine (Kyn) catalyzed by indoleamine 2,3-dioxygenase (IDO) (68). T cells can regulate Tumor repopulating cells (TRC) that can induce CD8+ T cells to express PD-1 through Kyn uptake and AhR activation through IDO-Kyn-AhR pathway (68). Besides, glutaric acid, a key metabolite produced during the breakdown of tryptophan and lysine within T cells, regulates T cell differentiation by altering epigenetic and energy metabolism (69).

### Fatty acid metabolic pathway

**Figure 1:**
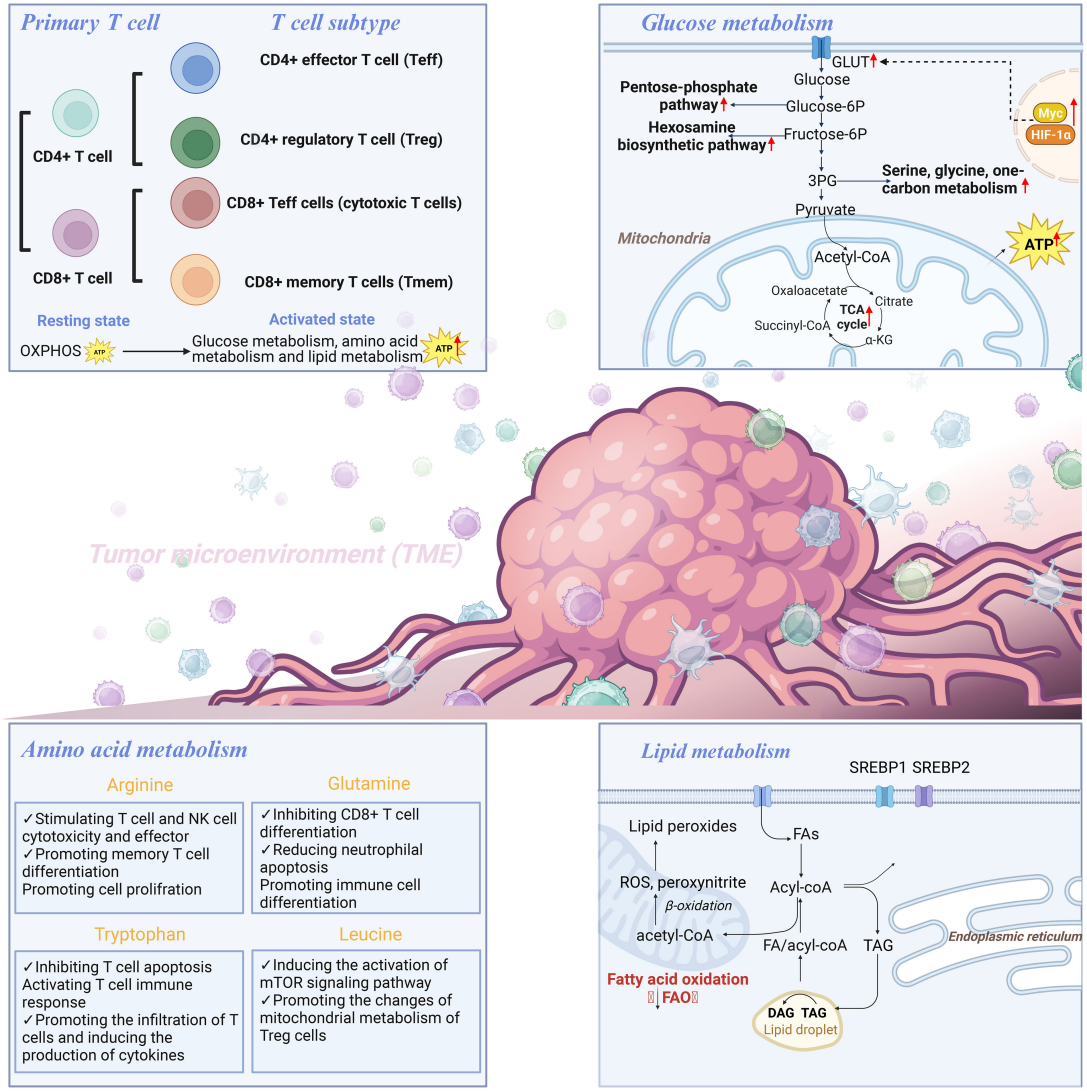
T cell hypermetabolism includes glucose metabolism, amino acid metabolism, and lipid metabolism.

Lipids are divided into a variety of subtypes based on their structural/biological characteristics, including fatty acids (FAs), phospholipids, cholesterol, triglycerides, etc. In TME, lipid metabolism, as an important energy source and one of the significant components of cell membrane, is closely related to T cell proliferation and function (70). T cell senescence driven by malignant tumor cells and TreGs is a common feature in cancer development. Senescent T cells have active glucose metabolism, but their lipid metabolism is unbalanced (71). Through experiments in mouse models of melanoma and breast cancer, Liu X found that the inhibition of phospholipase A2-IVA can reprogram lipid metabolism of effector T cells and prevent T cell aging. Oxidized cholesterol is enriched in the tumor microenvironment, and oxidized cholesterol inhibits SREPB2 pathway from activating LXR pathway, thus resulting in cholesterol deficiency and T cell dysfunction (72). Moreover, FAO is involved in the generation and functional maintenance of Tmem cells. Saibil et al. proved that, as the energy source, FAO enables Tmem cells to respond to antigen stimulation in a timely manner, which is conducive for maintaining the normal function of Tmem mitochondria and long-term cell survival (73). FAO regulates the balance between Teff and Treg cells (74). However, it should not be ignored that while Tregs do depend on mitochondrial function, it is not necessarily fatty acid oxidation. For example, Chapman et al. discovered that mTOR plays an important role in coordinating transcriptional and metabolic programs in activated treg subpopulations, thereby mediating tissue homeostasis processes (75). Weinberg et al. demonstrated that Treg cells require mitochondrial complex III to maintain immune regulatory gene expression and suppressive function (76). Furthermore, mitochondrial transcription factor A (Tfam) is essential for mitochondrial respiration and the control of mitochondrial DNA replication, transcription, and packaging. The loss of Tfam in Treg affects Treg homing and stability, thus leading to tissue inflammation in colitis, but enhances tumor rejection, which reveals the key role of TFAM-mediated mitochondrial respiration in regulating inflammation and anti-tumor immunity in Tregs (75, 77). Saravia et al. revealed that the transcriptional regulatory factor c-Myc can coordinate immune homeostasis by coordinating treg accumulation, transitional activation, and metabolic programming (78). However, in fact, several recent studies showed that Cpt1a is dispensable for Treg generation or function *in vivo*. For example, Raud et al. illustrated that the ACC2/Cpt1a axis is largely indispensable for the formation of Teff, Tmem or Tregcell, and the effects of etomoxil on T cell differentiation and function are independent of Cpt1a expression (79). This finding provides data support that metabolic pathways other than LC-FAO can promote Tmemor treg differentiation. Besides, Treg-specific deletion of Cox10, rather than FAO’s rate-limiting enzyme Cpt1a, has been confirmed to promote oxidative phosphorylation, thereby leading to impaired treg function and maturation (78). What’s more, the lipid metabolism reprogramming of CD8+ T cells can affect the biology of tumor cells (80). Yang W et al. identified cholesterol esterification enzyme (ACAT1) as a metabolic checkpoint that regulates tumor immune response, and inhibition of its activity can increase the level of free cholesterol on CD8+ T cell membrane, thereby enhancing the tumor-killing ability of CD8+ T cells (81). The main mechanism is that the plasma membrane cholesterol level of CD8+ T cells increases significantly, which helps the efficient formation of T cell antigen receptor clusters and immune synapses. Moreover, studies have confirmed that the combination of ACAT inhibitor Avasimibe and PD-1 antibody can further improve the effectiveness of tumor immunotherapy. Moreover, Fan J et al. found that mitochondrial ACAT1 and SIRT3 are upstream acetyltransferase and deacetylase of PDHA1 and PDP1, respectively, and knockdown of ACAT1 can weaken tumor growth (82). These studies suggest the potential of ACAT1 as a target for anti-cancer drugs. Overall, different states of T cells are controlled by multiple metabolic programs (83, 84) (**Figure 1**). The metabolic reprogramming of T cells is an essential component of the immune response. Understanding and manipulating T cell energy metabolism has significant implications for developing novel immunotherapies and enhancing immune responses against tumors.

## Various factors associated with T cell energy metabolism

**Figure 2:**
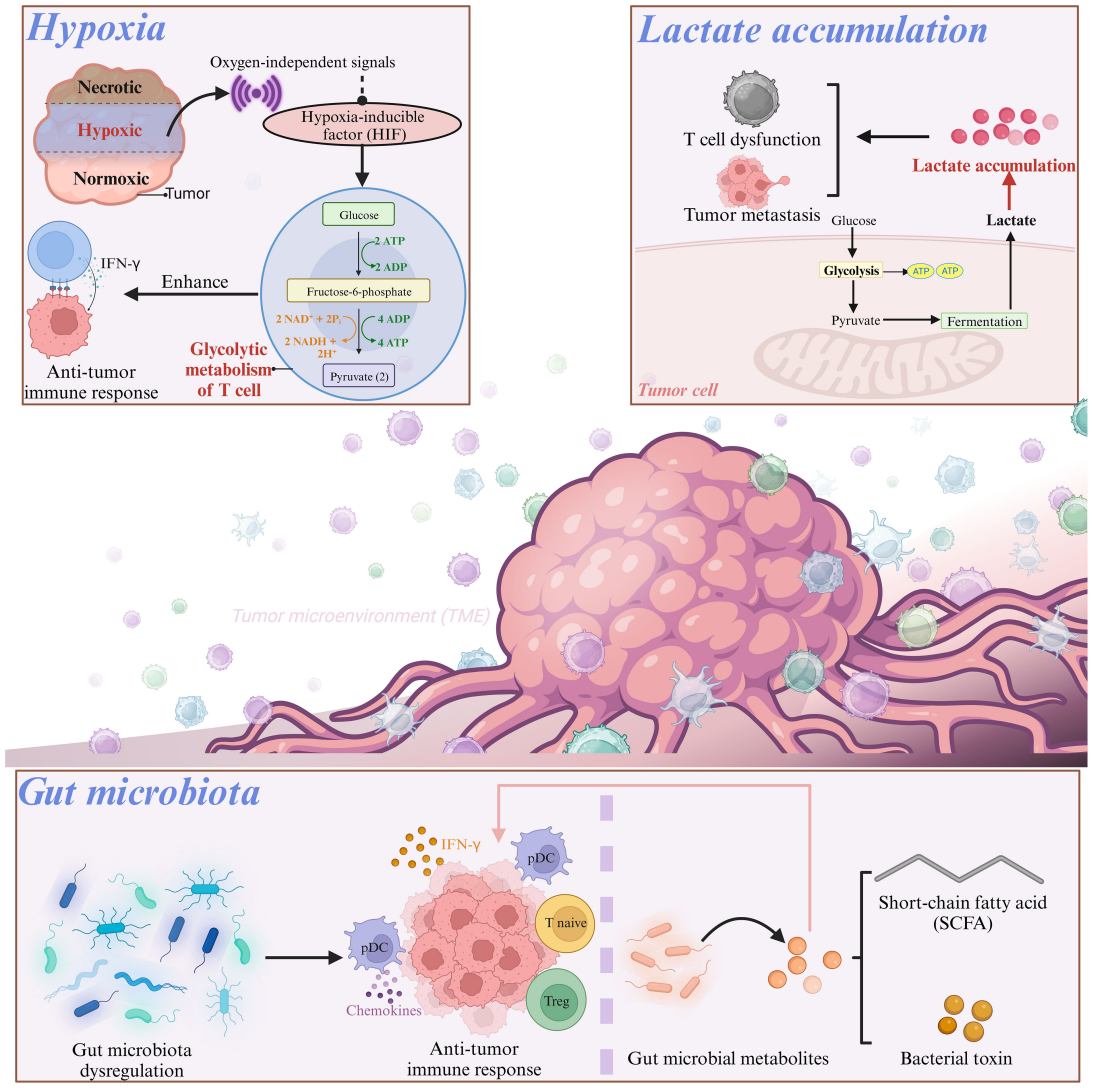
Various factors associated with T cell energy metabolism.

Research has shown that various factors associated with T cell energy metabolism have significant impacts on T cell function. Herein, T cell energy metabolism-related factors are shown in **Figure 2**.

### Hypoxia

Hypoxia in the TME has a unique two-sided effect on immune cell function (85). Under hypoxic conditions, multiple oxygen-independent signals, including those present in T cells, increased hypoxia-inducible factor (HIF) activity (86). After activation via oxygen-independent pathways, T cells depend on HIFs to sustain glycolytic metabolism. For instance, Oestreich et al. reported that following T cell activation, decreased interleukin (IL)-2 exposure lead to elevated B-cell lymphoma 6 (Bcl-6) expression, which directly inhibited glycolytic genes regulated by HIF-1α and c-myc. This mechanism suppresses glycolytic metabolism and effector function, and consequently influences the fate of T cells in a microenvironment-dependent manner (87). A previous investigation on Foxp3-regulatory CD4+ T cells revealed the involvement of HIF-1α in maintaining a glycolytic metabolic shift in CD4+ T cells, when it was activated and cultured *in vitro* with IL-27 (88), which suggested the crucial role of HIF-1α in modulating CD4+ T cell metabolism. The HIF-1α expression in CD8+ T cells promotes glycolytic metabolism, thus leading to enhanced proliferation and effector functions of CD8+ T cells (44, 89). Compared with normoxic T cells, hypoxic CD8+ T cells increased the packaging of granzyme B into particles and cleared B16 tumors more effectively in mice (90). This adaptive metabolic transformation and functional enhancement contribute to the anti-tumor effects of CD8+ T cells in a hypoxic environment. In a CRC cell model, it has been observed that hypoxia enhances the CD8+ T cell activity and promotes IFN-γ expression (91).

### Lactate accumulation

To achieve “metabolic equilibrium,” tumor cells not only consume important nutrients but also produce toxic metabolic waste to further impact the differentiation of CD8+ T cells and impair T cell function. Among these, lactate accumulation has attracted considerable attention. Owing to the aerobic glycolytic activity of tumor cells, lactate accumulates in the TME, and high lactate concentrations directly regulate the effector function of CD8+ T cells (92). Activated T cells primarily produce ATP and biomacromolecules via aerobic glycolysis. To maintain a high glycolytic rate, CD8+ T cells export lactate through Monocarboxylate Transporter 1 (MCT1). The transport activity of MCT1 is mainly regulated by the lactate concentration gradient in the cell membrane. However, lactate accumulation in the TME disrupts MCT1-mediated lactic acid export from CD8+ T cells, and further induces an inhibitory tumor-infiltrating (TIL) CD8+ phenotype (92). Robert et al. also demonstrated that the build-up of lactic acid generated by CRC cell metabolism and the resulting acidic environment could repress glycolysis and impair T cell functionality (93). Additionally, lactic acid can induce the upregulation of CXCL10, which facilitates the attraction of CD4+ T cells to the metastatic site and triggers receptor activator of nuclear factor-κB-ligand (RANKL) production, thereby promoting CRC bone metastasis (94, 95).

### Gut microbiota

Dysregulation of the gut microbiota is a crucial factor in both cancer onset and immune system modulation, which ultimately affectes the efficacy of immunotherapy (96). In mouse models, a microbial mixture could boost anticancer immunity by stimulating the production of IFN-γ-producing cytotoxic T cells (CTCs) within tumor tissues (97). *Escherichia coli, Firmicutes, and Bacteroides fragilis* have been found to promote the migration of T cells into CRC tissues by upregulating the expression of chemokines involved in T cell recruitment (98). However, colibactin-producing *Escherichia coli* can impair the infiltration of CD3+ and CD8+ T cells into the CRC, thus resulting in tumor resistance to immunotherapy (99). Moreover, the colonization of Bifidobacterium in the intestine can result in changes in the composition of the gut microbiota and increase the suppressive function of Treg cells by promoting mitochondrial activity, which indicates the crucial role of gut microbiota in the metabolic reprogramming of T cells (100).

What’s more, the gut microbiota generates multiple metabolites that can interact with host tissues and the immune system, which exertes a significant influence on T cell development and function (101, 102). Intestinal immune homeostasis can be maintained by Treg cells, and microbial bile acid metabolites are vital for generating colonic Treg cells and suppressing intestinal inflammation (103–106). Bacterial production of short-chain fatty acids (SCFAs), such as butyrate, can alleviate dextran sulfate sodium and clostridium difficile induced colitis by preventing Th17 through activation of SIRT1/mTOR (107). SCFAs, as the substrate for β-oxidation, can elevate the mitochondrial mass and glucose transporter protein type 1 (GLUT1) expression as well as stimulate OXPHOS and glutaminolysis instead of glycolysis in activated CD8+ T cells. Not only that, butyrate can promote cell metabolism and enhance the memory potential of activated CD8+ T cells. In this process, butyrate decoups the tricarboxylic acid cycle from glycolytic input to CD8+ T cells, which prefertionally provides fuel for oxidative phosphorylation through ongoing glutamine utilization and fatty acid catabolism (83). SCFAs, as the substrate for β-oxidation, can elevate the mitochondrial mass and glucose transporter protein type 1 (GLUT1) expression as well as stimulate OXPHOS and glutaminolysis instead of glycolysis in activated CD8+ T cells (108). The gut microbiota-derived metabolite butyrate modulates the effector function of CD8+ T cells (83). Additionally, pentanoate, a subdominant type of SCFA, was found to directly affect CD4+ T cell metabolism. Pentanoate stimulated glycolysis and increased acetyl-CoA levels in Th17 cells (109). Isoallolithocholic acid (isoalloLCA), a secondary bile acid, augments OXPHOS in CD4+ T cells, which is indicated by the elevated oxygen consumption and mitochondrial membrane potential (104). As a key metabolite produced by glycolytic metabolism of glucose molecules, lactic acid prevents the upregulation of activated T nuclear factors in T cells and NK cells, thus resulting in reduced IFN-γ production. As a potent inhibitor of T and NK cell function and survival, lactic acid can cause tumor immune escape (110). Besides, lactate can increase the dryness of CD8+T cells and enhance anti-tumor immunity (111). In addition, studies have shown that lactic acid is an active checkpoint for Treg cell function, and it can up-regulate PD-1 expression in highly glycolytic TME (112). Treg cells up-regulate pathways involved in the metabolism of lactic acid. Lactate uptake is essential for the function of peripheral treg cells, but it requires intratumoral uptake, thereby resulting in slower tumor growth and increased response to immunotherapy (50).

## T-cell energy metabolism in cancer immunotherapy

T cells are central players in mounting an effective anti-tumor immune response. Activated T cells must adjust their metabolism to meet the energy requirements associated with rapid proliferation and effector function. Therefore, targeting T cell metabolism is a promising strategy in the development of T cell-based immunotherapeutics. Current T cell-based immunotherapeutics mainly include ICIs and autologous T cell therapies.

## ICIs

Immune checkpoints are crucial elements of the immune system that contribute to the maintenance of immune homeostasis by controlling the type, intensity, and duration of immune responses. Immune checkpoints are ligand–receptor complexes that modulate immune responses by transmitting costimulatory or inhibitory signals. The overexpression of immune checkpoints in cancer are linked to T cell exhaustion, thus leading to dysfunctional T lymphocytes that exhibit decreased effector function and impaired proliferation (113). Among the numerous tumor-associated immune checkpoints, CTLA-4 and PD-1 are two critical molecules, and targeting them has demonstrated effectiveness in promoting the activation of anti-tumor immune responses (114). ICIs are monoclonal antibodies (mAbs) that block immune checkpoint proteins expressed on immune cells, such as T cells and tumor cells, thereby releasing the brakes on the immune system and enabling the elimination of tumor cells (115). mAbs that specifically target co-inhibitory immune checkpoints, such as PD-1 and CTLA-4, have demonstrated clinical effectiveness in various types of cancers, including CRC (116, 117). However, ICIs have not shown any clinical benefit or improvement in survival in patients with non-mismatch repair deficient (dMMR)/microsatellite instability-high (MSI-H) metastatic colorectal cancer (mCRC), accounting for 96% of the patients with metastatic disease (118, 119).

Additionally, immunosuppressive receptors alter the anti-tumor effects of T cells by interfering with their metabolism. It has been reported that PD-1 can affect the metabolic reprogramming of T cells by suppressing glycolysis and elevating lipolysis and FAO (120), which suggested that immune checkpoint blockade can promote the metabolic reprogramming of Teff cells, thus enhancing their anti-tumor effect. Conversely, CTLA-4 inhibits glycolysis without augmenting FAO, which suggestes that CTLA-4 sustains the metabolic profile of non-activated cells. Collectively, PD1- and CTLA4-targted ICIs enhance effector T cell function via increasing glucose influx and glycolysis. Importantly, the extrinsic metabolic barriers on tumor-infiltrating immune cells caused by the TME, such as lactate accumulation, hypoxia, and mitochondrial dysfunction, can be overcome by ICIs (121). The anti-PD1 blockade and bezafibrate combination treatment has been shown to promote mitochondrial biogenesis, FAO, and OXPHOS in CD8+ T cells (122). Therefore, targeting T cell metabolism represents a promising approach for the improvement of the outcomes of ICIs.

## Autologous T cell therapies

Autologous T cell therapies mainly employ CAR- and T cell receptor (TCR)- based therapies that recognize antigens on targeted tumor cells and offer treatment options for cancer (123, 124). CAR-T cells are engineered T cells that express synthetic T cell receptors and enable them to recognize and target tumor surface antigens. These engineered T cells redirect the polyclonal T cell response toward tumor cells, thus resulting in the eradication of tumors (125). Several CAR-T cell therapies have been used to treat hematological malignancies (126). However, some obstacles, such as the absence of suitable target antigens and an immunosuppressive TME, hinder their clinical application (127). TCR-T cells can recognize antigens expressed on both the cell surface and within intracellular compartments, thus making it a compelling avenue for solid tumor treatment (128). Interestingly, the metabolic reprogramming of T cells through gene editing can help T cells resist unfavorable environmental factors in TME. The mitochondrial regulator PPARGC1A(peroxisome proliferator activating receptor γ, coactivator 1α) is often described as a major regulator of mitochondrial biogenesis and a central player in regulating antioxidant defense, capable of reducing aging in vascular smooth muscle cells (VSMCs) (129). However, there is growing evidence that PGC-1α is also involved in complex regulation of mitochondrial mass beyond biogenesis, including mitochondrial network dynamics and autophagy clearance of damaged mitochondria. Furthermore, it was found that the deficiency of PGC-1α can cause the loss of mitochondrial function of Teff cells, and increasing the expression of PGC-1α can save the mitochondrial function of Teff cells and increase the production of cytokines, thus enhancing the anti-tumor effect of T cells (130). Moreover, lysine demethylase 3A (KDM3A) can bind to PGC-1α and demethylate the monomethyl lysine (K) 224 of PGC-1α under normal oxygen conditions. Up-regulated expression of PGC-1αK224R mutant can promote mitochondrial biogenesis, reactive oxygen species (ROS) production and tumor cell apoptosis in mice under hypoxia, and inhibit the growth of brain tumors (131). Furthermore, the study found that cholesterol esterification enzyme ACAT1 is also a good regulatory target in the metabolic pathway of T cells. Knockout or inhibition of acetyl-coacetyltransferase (ACAT1) can increase the cholesterol concentration of CD8+ T cell membrane, so that this type of killer T cells can be more easily polymerized than the original and better form immune synapses, thereby improving antigen sensitivity and improving immune efficacy (81). Moreover, studies have confirmed that the combination of ACAT inhibitor Avasimibe and PD-1 antibody can further improve the effectiveness of tumor immunotherapy. In addition, Fan J, et al. discovered that ACAT1 mainly sends signals through PDP1 and PDHA to promote Warburg effect and tumor growth. When ACAT1 is knocked down, tumor growth is inhibited. These results also suggest the potential of ACAT1 as a target for anti-cancer drugs (82). In conclusion, metabolic remodeling of T cells through gene editing or transduction of specific genes *in vitro* can enhance the antitumor properties of T cells. The development of new strategies in improving the metabolic fitness of autologous T cells can improve the therapeutic efficacy of anti-tumor immunity.

Furthermore, TGF-beta has gradually been found to play an important role in T cell energy metabolism and cancer immune evasion. Checkpoint blocking drugs represented by PD-1/PD-L1 antibodies have achieved good results in the treatment of a variety of cancers. However, the overall efficiency is still not high. Among them, the heterogeneity of tumor and the diversity of immune microenvironment are the main reasons limiting the therapeutic effect. Recent studies have revealed that anti-TGF-β/PD-L1 bispecific antibody YM101 can simultaneously block the PD-1/PD-L1 and TGFR2/TGF-β signaling pathways, promote the activation of efficient T cells, regulate the tumor microenvironment, and reverse immunosuppression and fibrosis (132). Meanwhile, its anti-tumor effect superior to PD-L1 monoclonal antibody has been verified in a variety of mouse tumor bearing models. In addition, Yi M et al. also constructed a bispecific antibody (called BiTP) against TGF-β and human PD-L1 and found that BiTP retained the binding affinity and biological activity of the parent antibody, and it had strong anti-tumor activity against the parent antibody in triple-negative breast cancer (TNBC) (133). These studies indicate that new drugs based on TGF-β/PD-L1 double antibodies are expected to provide a new solution to the existing immunotherapy dilemma, and have important clinical significance and translational value.

## Conclusion

Metabolic reprogramming is the metabolic change that cells undergo in response to various stressors. Similar to tumor cells, activated T cells in the TME undergo metabolic reprogramming to acquire energy, and the failure of activated T cells to obtain sufficient nutrients or undergo metabolic re-wiring can damage their effector function. Factors, such as hypoxia, lactate accumulation, and the gut microbiome, may affect T cell activation and energy metabolism. Targeting T cell metabolism is a potentially promising approach for eliciting durable immune responses in tumors that are resistant to conventional immunotherapies. Therefore, therapeutic approaches targeting T-cell metabolism may offer an opportunity to optimize and improve current anti-tumor therapeutic strategies.

## Author contributions

LX: Writing – original draft. SY: Writing – original draft. CJ: Writing – original draft, Validation. SYF: Validation, Writing – original draft. WX: Writing – original draft, Visualization. ZW: Writing – original draft. HS: Writing – original draft. YX: Writing – original draft.
